# Supplementary figures and images for: HIV impairs and exploits pulmonary Th17 and Th22 cell-mediated immune responses to Mycobacterium tuberculosis
Source: PLoS Pathog. 2026 Jan 30;22(1):e1013897. doi: 10.1371/journal.ppat.1013897 (PMC12872012; doi:10.1371/journal.ppat.1013897)

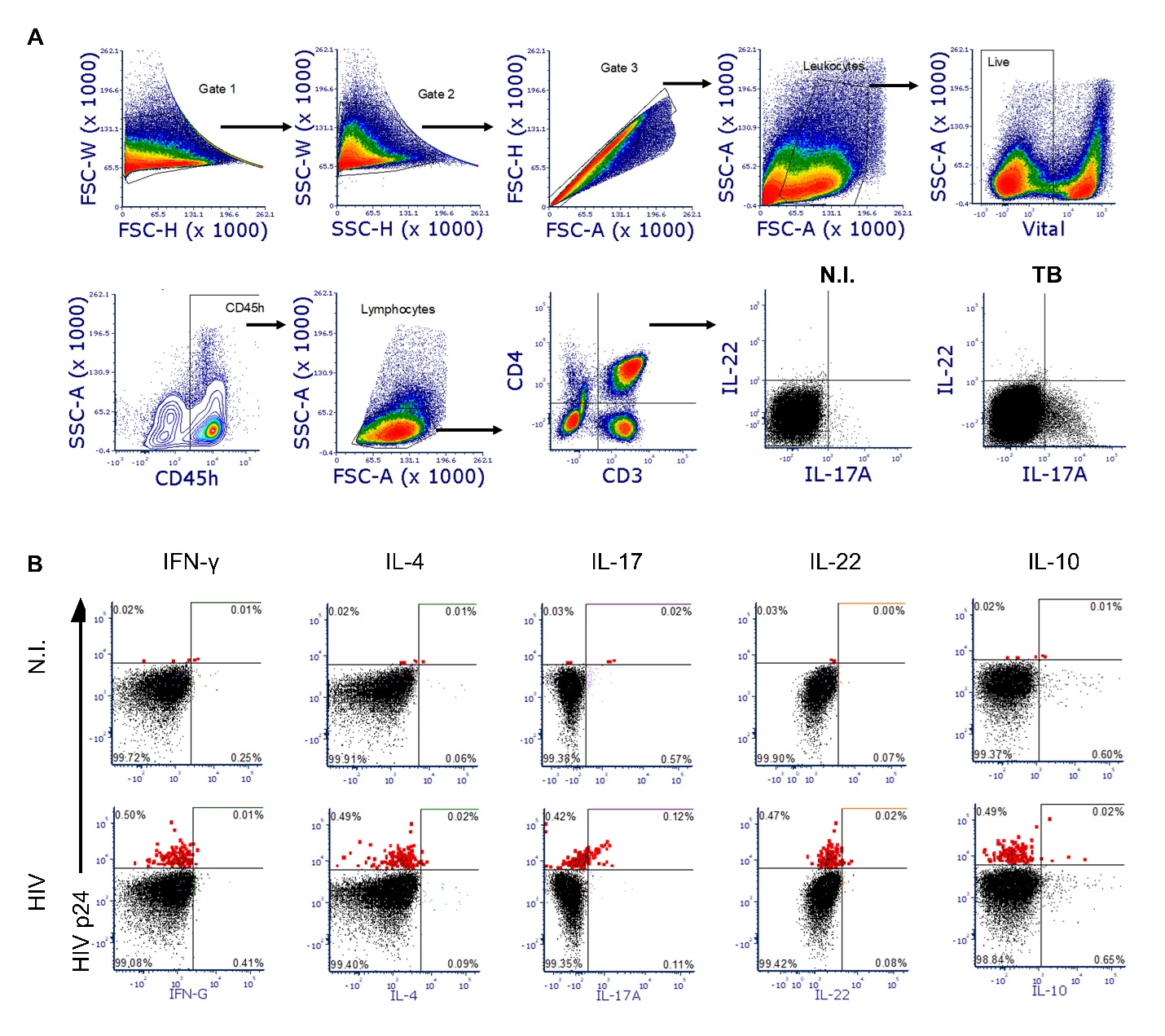

Supplement: S1 Fig — (A) Gating used for selection of lung and spleen populations in both experiments. Sequential gating combinations of FSC-A, FSC-H, FSC-W, SSC-H, and SSC-W selected singlets. Among singlets (gate 3), Leukocytes were selected by size (FSC-A) and granularity (SSC-A). Viable (vital, negative for Live/dead Near IR) human CD45 + cells (leukocytes) were further gated for human macrophages (CD68 + CD14-). Among leukocytes, lymphocytes were selected for T cells (CD3+), and T helper (CD3 + CD4+) markers, which were additionally assessed for intracellular cytokines. In the example graphic, Th17 and Th22 are assessed by IL-17 and IL-22 intracellular markers, respectively, shown in the right panel of Fig A within a representative uninfected and infected mouse. (B) Representative gating used in spleen for identification of HIV in the different cytokine producer subsets in Fig 2E and 2H among spleen controls (N.I., non-infected) (top) and HIV-infected spleen samples at 14 dpi (bottom). (TIF) [file ppat.1013897.s001.tif]

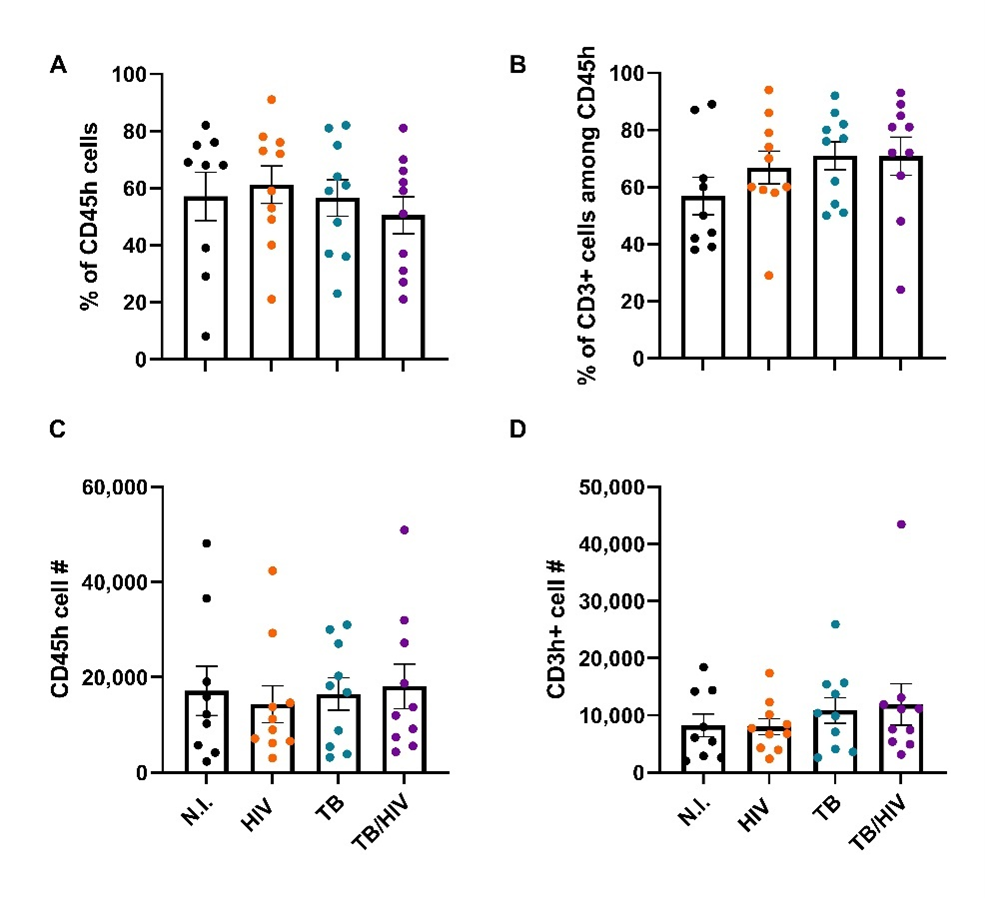

Supplement: S2 Fig — HIS mice were equally separated into four groups of 9–10 mice/group. Retroorbital blood was taken to assess distribution of human responses by flow cytometry before aerosol Mtb infection (or day 0) as in Fig 3A. (A) Reconstitution percentage, evaluated as % of human CD45+ (leukocyte) cells, among total leukocytes, using mouse and human CD45 markers. (B) Percentage of human T cells (CD3+), among the total human CD45 + leukocyte population. (C) Human CD45 leukocyte cell count. (D) Total human CD3 + cell count before infection among the four experimental groups: Non-infected, HIV, TB, and TB-HIV. No statistical difference was found before the infection (One-Way ANOVA with Benjamini FDR correction). (TIF) [file ppat.1013897.s002.tif]

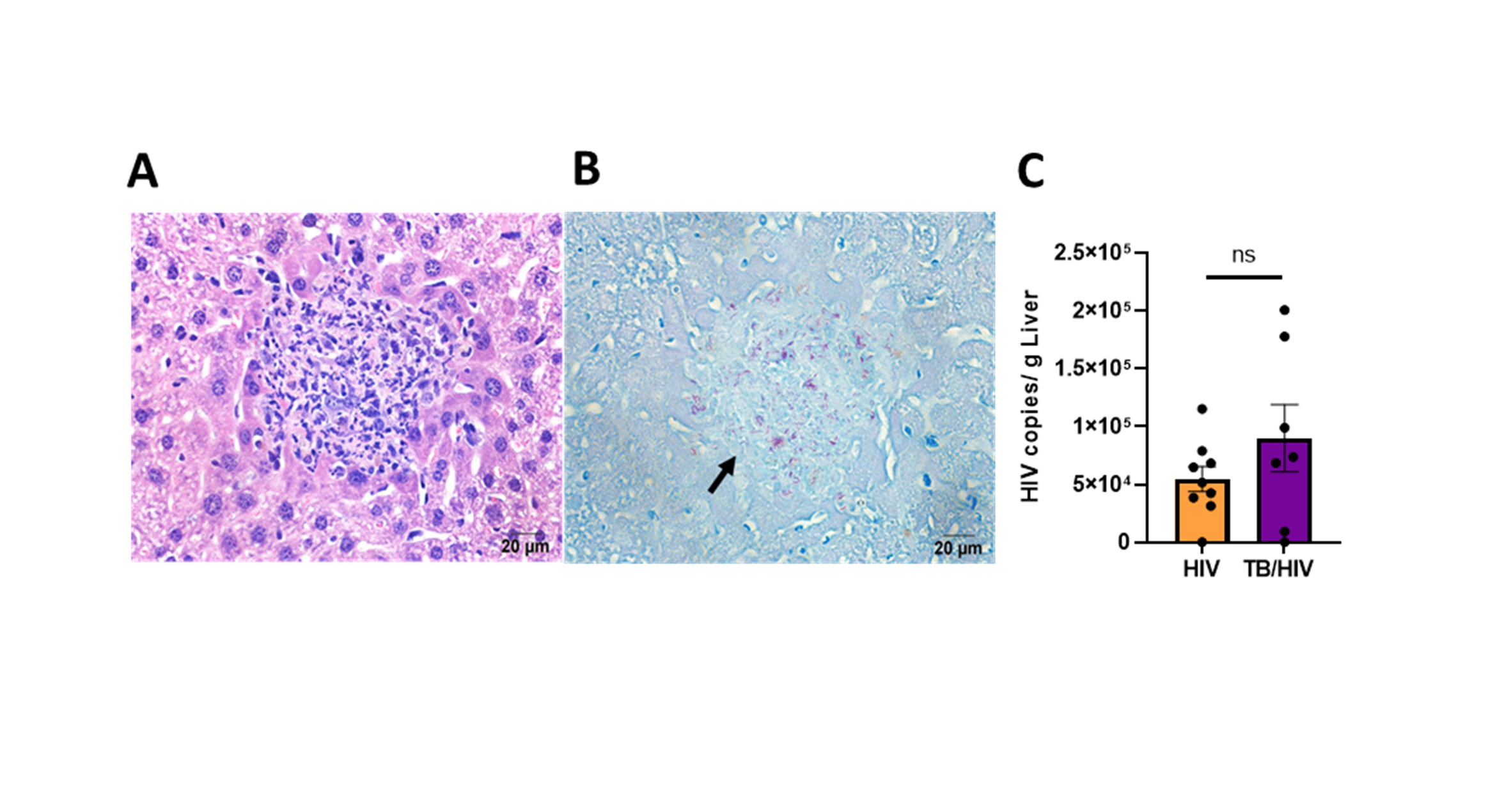

Supplement: S3 Fig — (A) Representative H&E image of Mtb-infected liver, showing a granulomatous lesion. (B) Same histological area (arrow), with bacilli observed by AFB (magenta) staining. (C) HIV viral copies per gram of liver as detected by using RT-PCR. Ns = non-significant result based on Student’s T-test. (TIF) [file ppat.1013897.s003.tif]

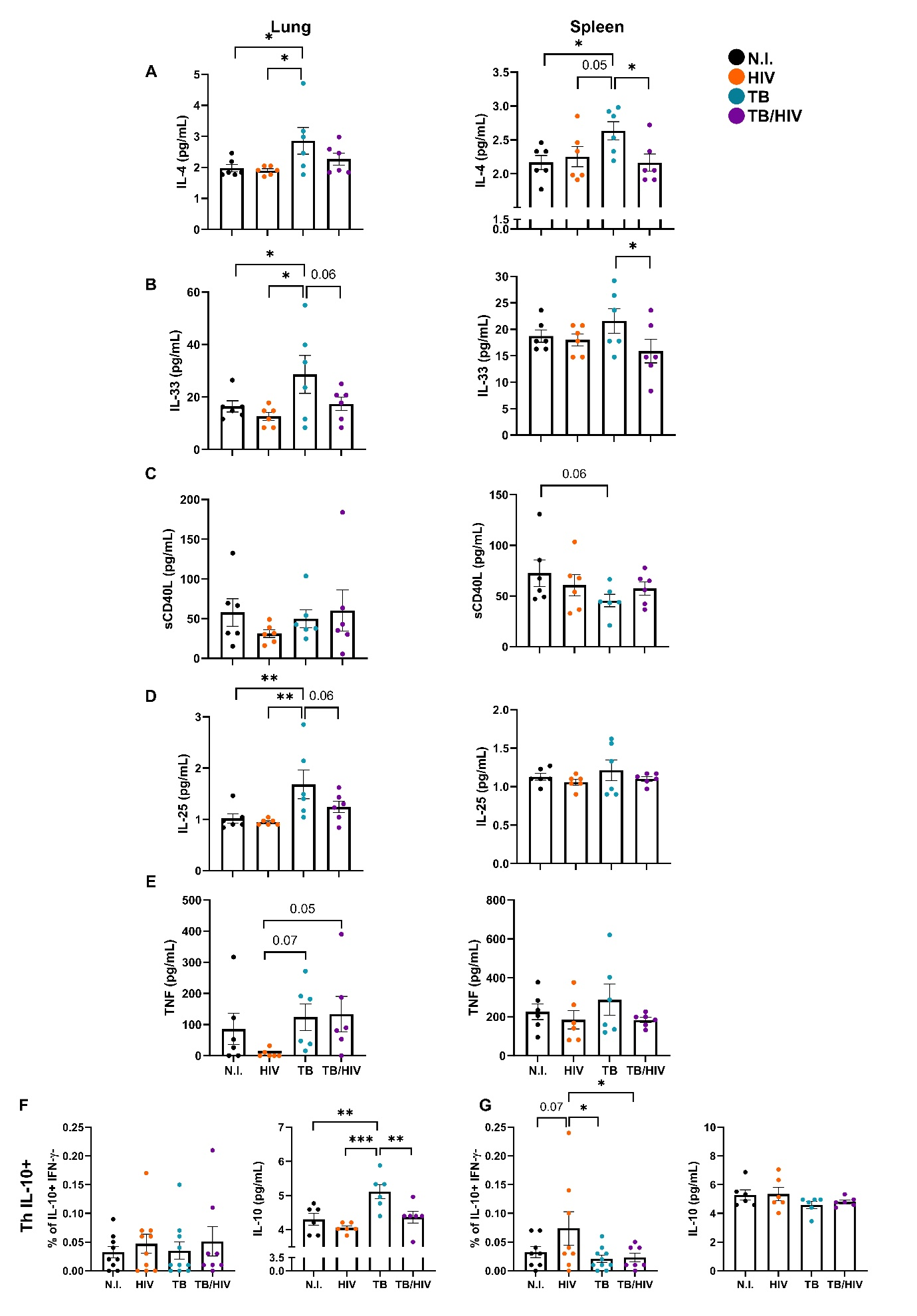

Supplement: S4 Fig — Supernatants were collected after cellular disaggregation for flow cytometry, frozen, and irradiated. After protein concentration, cytokines were quantified in supernatants as pg/mL by multiplex ELISA. In A-E we observe cytokines in lung (left panels) and spleen (right panels) including (A) IL-4, (B) IL-33, (C) soluble CD40 ligand, (D) IL-25 and (E) TNF in the four experimental groups: non-infected, HIV, TB, and TB-HIV groups. (F-G) Percentage of Th IL-10+ (IL-10 + IFNγ-)(left), or IL-10 cytokine production (right) in lung (F) or spleen (G). *p < 0.05, **p < 0.01, ***p < 0.001, (One-Way ANOVA with Benjamini FDR correction). (TIF) [file ppat.1013897.s004.tif]

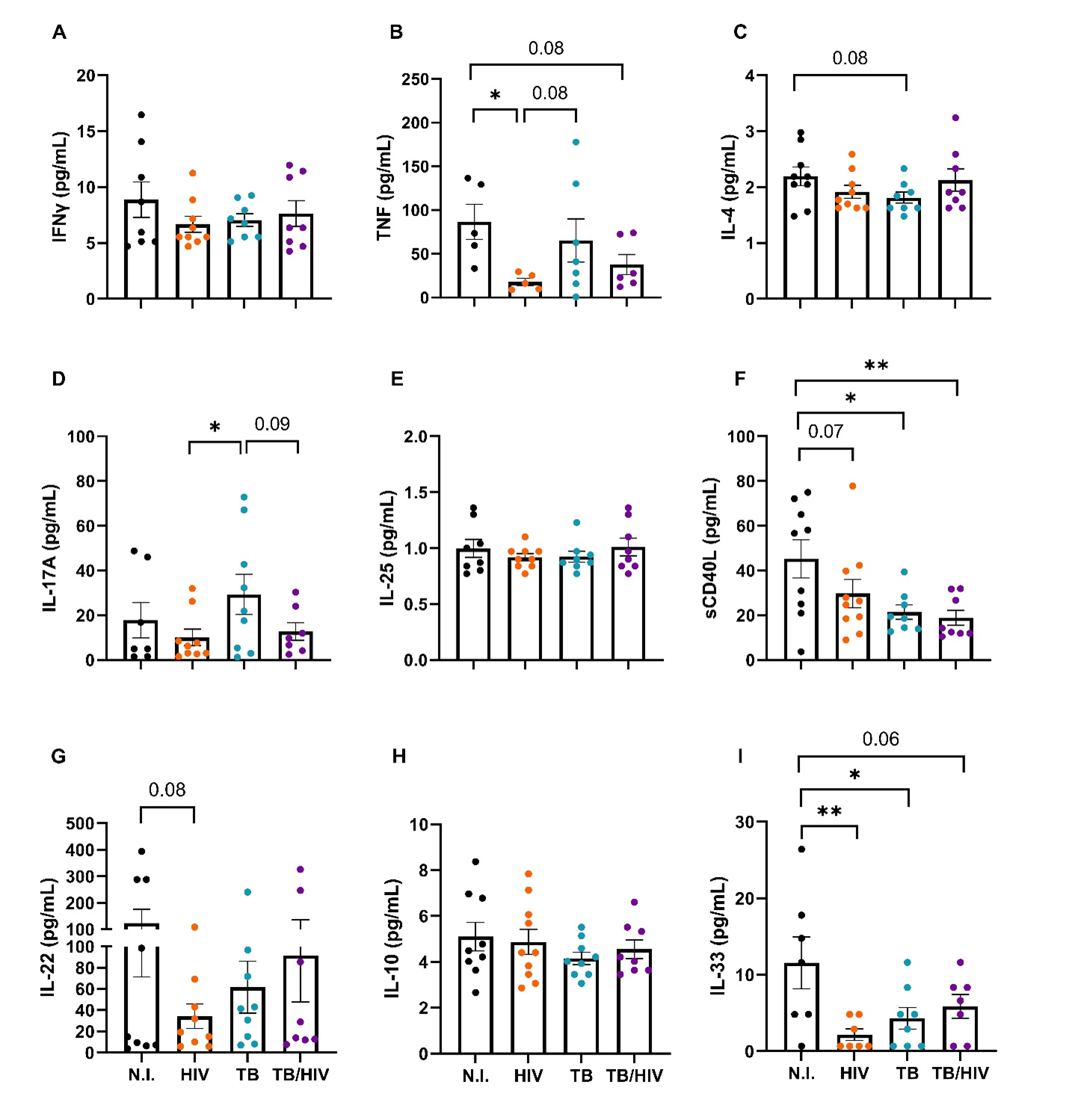

Supplement: S5 Fig — Plasma samples were obtained after terminal intracardiac puncture, frozen, irradiated and cytokines were measured by multiplex ELISA. The different experimental groups are depicted with different color dots. From left to right, non-infected, HIV, TB, and TB-HIV. Plasma cytokines quantified in pg/mL were (A) IFNγ, (B) TNF, (C) IL-4, (D) IL-17A, (E) IL-25, (F) soluble CD40 ligand, (G) IL-22, (H) IL-10 and (I) IL-33. *p < 0.05, **p < 0.01, (One-Way ANOVA with Benjamini FDR correction). (TIF) [file ppat.1013897.s005.tif]

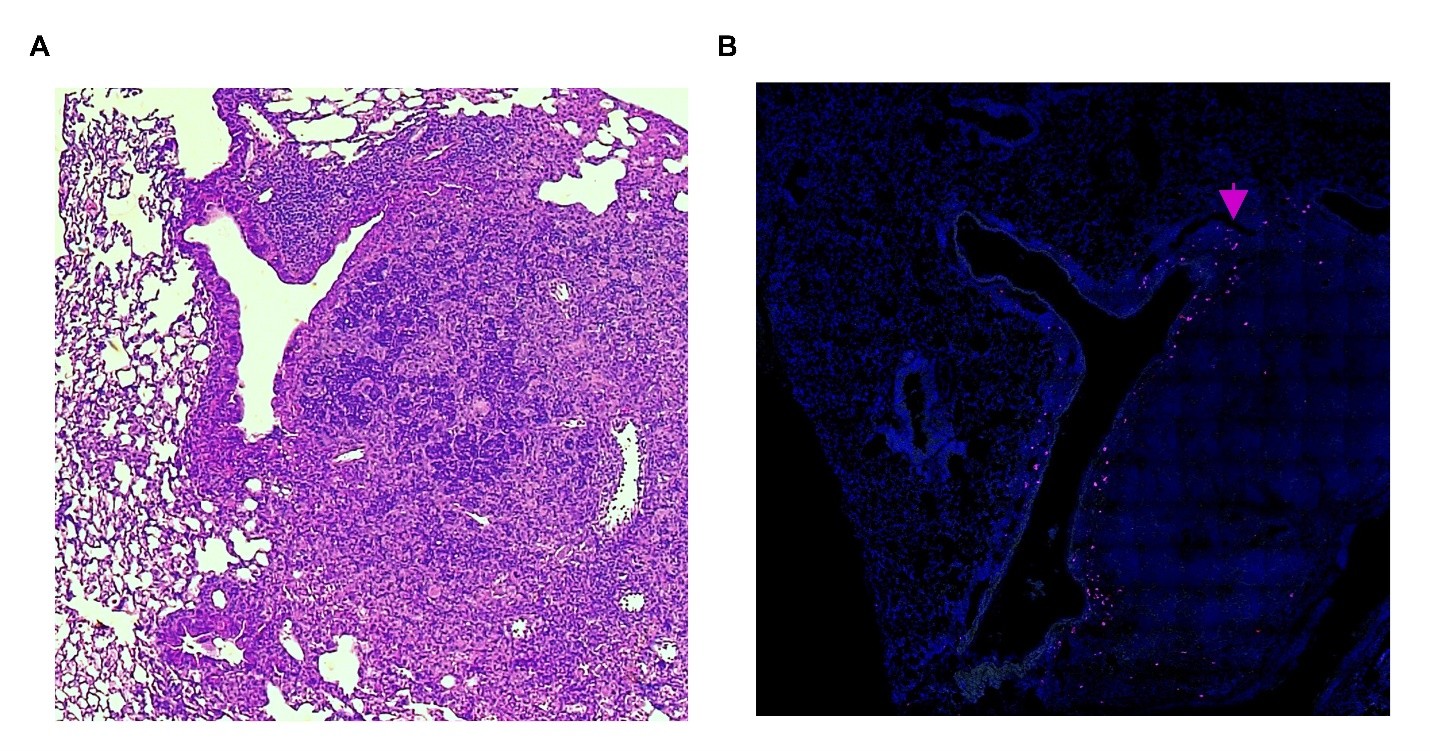

Supplement: S6 Fig — (A) Organ scan overviews of lung in the TB-HIV group by H&E or (B) the respective tissue section RNAscope analysis , depicting HIV in cells surrounding the periphery of granulomas and the peribronchial areas of the lung by 9 days of HIV co-infection. In B, the representative immunofluorescent RNAscope image was stained through in situ hybridization, detecting RNA of HIV (non-gag-pol) with OPAL 690 (purple). (TIF) [file ppat.1013897.s006.tif]

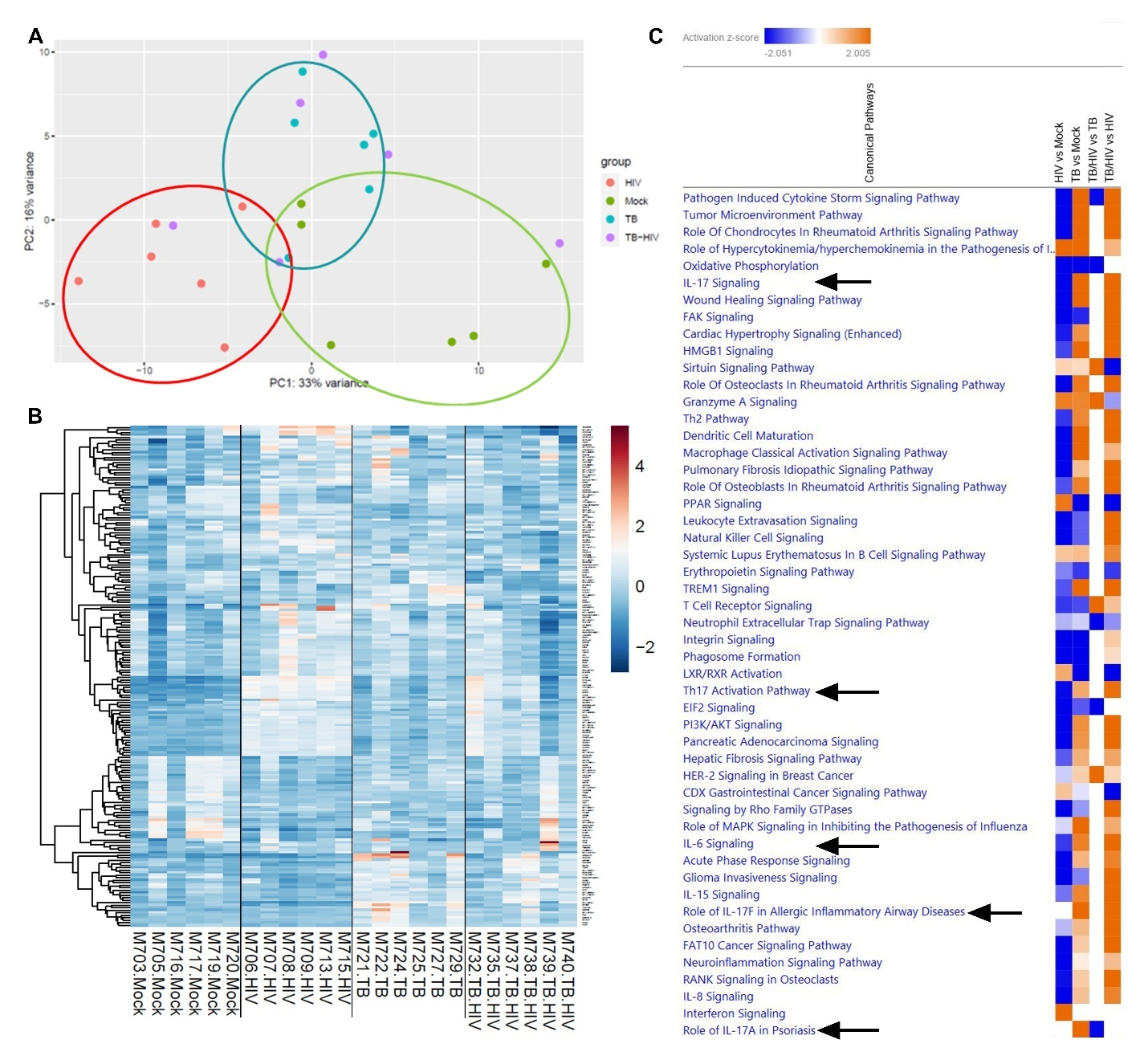

Supplement: S7 Fig — (A) Principal component analysis clusters the human lung responses according to infection in the HIS mouse model. Each dot represents an infected animal with HIV, Mtb, Mtb-HIV, or uninfected. (B) Heat map to observe the top 200 variable genes across all the samples, depicting transcriptome changes in the lungs. (C) Principal 50 canonical pathways affected by comparison of the four experimental groups, with arrows indicating IL-17 related pathways. The predictions were calculated through Ingenuity Pathway Analysis. Blue color depicts a negative activation z-score, predicting downregulation of the pathway. Orange color predicts upregulation by activation z-scores. n = 6/group. (TIF) [file ppat.1013897.s007.tif]

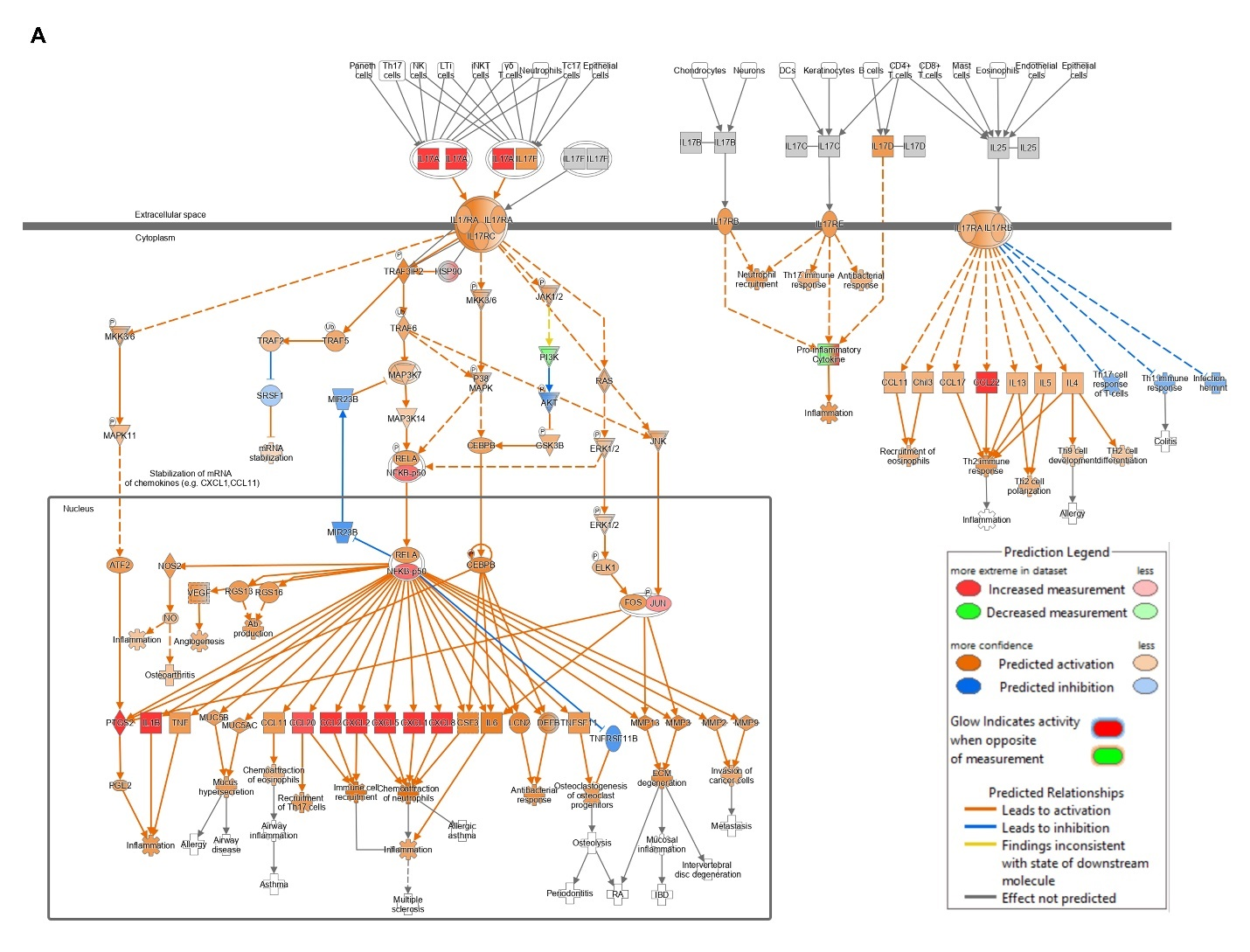

Supplement: S8 Fig — (A) Signaling pathway comparison of TB versus non-infected. Ingenuity Pathway Analysis predicts activation (orange) for most of the IL-17 signaling pathway due to observations of experimentally upregulated genes (red) in RNA-seq analysis. (TIF) [file ppat.1013897.s008.tif]
